# Development of a CT number calibration audit phantom in photon radiation therapy: A pilot study

**DOI:** 10.1002/mp.14077

**Published:** 2020-02-29

**Authors:** Minoru Nakao, Shuichi Ozawa, Hideharu Miura, Kiyoshi Yamada, Kosaku Habara, Masahiro Hayata, Hayate Kusaba, Daisuke Kawahara, Kentaro Miki, Takeo Nakashima, Yusuke Ochi, Shintaro Tsuda, Mineaki Seido, Yoshiharu Morimoto, Atsushi Kawakubo, Hiroshige Nozaki, Yasushi Nagata

**Affiliations:** ^1^ Hiroshima High‐Precision Radiotherapy Cancer Center 3‐2‐2, Futabanosato, Higashi‐ku Hiroshima 732‐0057 Japan; ^2^ Department of Radiation Oncology Graduate School of Biomedical & Health Sciences Hiroshima University 1‐2‐3 Kasumi, Minami‐ku Hiroshima 734‐8551 Japan; ^3^ Department of Radiation Oncology Hiroshima University Hospital 1‐2‐3 Kasumi, Minami‐ku Hiroshima 734‐8551 Japan; ^4^ Radiation Therapy Section Department of Clinical Support Hiroshima University Hospital 1‐2‐3 Kasumi, Minami‐ku Hiroshima 734‐8551 Japan; ^5^ Department of Radiology Hiroshima Prefectural Hospital 1‐5‐54, Ujinakanda, Minami‐ku Hiroshima 734‐8530 Japan; ^6^ Radiation Therapy Department Hiroshima City Hiroshima Citizens Hospital 7‐33, Motomachi, Naka‐ku Hiroshima 730‐8518 Japan; ^7^ Division of Radiology Hiroshima Red Cross Hospital & Atomic‐bomb Survivors Hospital 1‐9‐6, Senda, Naka‐ku Hiroshima 730‐8619 Japan

**Keywords:** audit, CT number calibration, photon radiation therapy, quality assurance, stoichiometric method

## Abstract

**Purpose:**

In photon radiation therapy, computed tomography (CT) numbers are converted into values for mass density (MD) or relative electron density to water (RED). CT‐MD or CT‐RED calibration tables are relevant for human body dose calculation in an inhomogeneous medium. CT‐MD or CT‐RED calibration tables are influenced by patient imaging (CT scanner manufacturer, scanning parameters, and patient size), the calibration process (tissue‐equivalent phantom manufacturer, and selection of tissue‐equivalent material), differences between tissue‐equivalent materials and standard tissues, and the dose calculation algorithm applied; however, a CT number calibration audit has not been established. The purposes of this study were to develop a postal audit phantom, and to establish a CT number calibration audit process.

**Methods:**

A conventional stoichiometric calibration conducts a least square fit of the relationships between the MD, material weight, and measured CT number, using two parameters. In this study, a new stoichiometric CT number calibration scheme has been empirically established, using three parameters to harmonize the calculated CT number with the measured CT number for air and lung tissue. In addition, the suitable material set and the minimal number of materials required for stoichiometric CT number calibration were determined. The MDs and elemental weights from the International Commission on Radiological Protection Publication 110 were used as standard tissue data, to generate the CT‐MD and CT‐RED calibration tables. A small‐sized, CT number calibration phantom was developed for a postal audit, and stoichiometric CT number calibration with the phantom was compared to the CT number calibration tables registered in the radiotherapy treatment planning systems (RTPSs) associated with five radiotherapy institutions.

**Results:**

When a least square fit was performed for the stoichiometric CT number calibration with the three parameters, the calculated CT number showed better agreement with the measured CT number. We established stoichiometric CT number calibration using only two materials because the accuracy of the process was determined not by the number of used materials but by the number of elements contained. The stoichiometric CT number calibration was comparable to the tissue‐substitute calibration, with a dose difference less than 1%. An outline of the CT number calibration audit was demonstrated through a multi‐institutional study.

**Conclusions:**

We established a new stoichiometric CT number calibration method for validating the CT number calibration tables registered in RTPSs. We also developed a CT number calibration phantom for a postal audit, which was verified by the performances of multiple CT scanners located at several institutions. The new stoichiometric CT number calibration has the advantages of being performed using only two materials, and decreasing the difference between the calculated and measured CT numbers for air and lung tissue. In the future, a postal CT number calibration audit might be achievable using a smaller phantom.

## 1 INTRODUCTION

Computed tomography (CT) images — required for contouring a target and calculating dose distribution in a patient's body — are imported into radiotherapy treatment planning systems (RTPSs). In photon radiation therapy, CT numbers are converted to mass density (MD), or relative electron density to water (RED), according to the applicable RTPS or the dose calculation algorithm, and the dose distribution is calculated for the human body in an inhomogeneous medium. Dose calculation in an inhomogeneous medium is influenced by four factors: patient imaging (itself influenced by CT scanner manufacturer, scanning parameters, and patient size), the calibration process (influenced by tissue‐equivalent phantom manufacturer and selection of tissue‐equivalent materials), the difference between tissue‐equivalent material and standard tissues, and the dose calculation algorithm applied. To validate dose calculation accuracy in the inhomogeneous medium, a comparison between the calculation and measurements, using lung or bone equivalent phantoms, is usually conducted.^1^, ^2^, ^3^, ^4^, ^5^ Final dose calculation results in the inhomogeneous medium vary according to the four factors above; however, practitioners such as radiation therapists or medical physicists can only adjust two of these, patient imaging and the calibration process. Therefore, it is valid to review patient imaging and the calibration processes using a third party; however, such patient imaging and calibration process reviews have not been performed.

Most radiotherapy institutions register the CT number calibration table in the RTPS, by scanning commercially available tissue‐equivalent material. The CT number calibration table is easily obtained using these tissue‐equivalent phantoms; however, tissue‐equivalent materials are determined by the manufacturer. A radiation therapist and medical physicist must create a consecutive CT calibration table from the discrete CT number for each tissue.^6^

It is appropriate to compare the CT calibration table with standard tissue data from the CT number calibration audits of a third party, simply because some tissue‐equivalent materials differ from standard tissues. A standard tissue’s CT numbers are calculated using a stoichiometric CT number calibration,^7^, ^8^ based on the MDs and elemental weights of the standard tissues, which are obtained from the International Commission on Radiological Protection Publication 23 (ICRP 23),^9^ the International Commission on Radiation Units and Measurements Report 44 (ICRU 44),^10^ ICRU 46,^11^ and ICRP 110.^12^ Therefore, stoichiometric CT number calibration based on standard tissue data is useful for CT number calibration audits by third parties.

Stoichiometric CT number calibration has three steps: a set of materials with known MDs and elemental weights are scanned with a CT scanner; relationships between the MDs, elemental weights, and measured CT numbers are determined with a multiparameter fit; and the theoretical CT numbers for standard tissues are calculated from the obtained fitting parameters and standard tissue data.

Authors of several studies have attempted to compare stoichiometric CT number calibration with a tissue‐substitute CT number calibration,^13^, ^14^, ^15^ and compare the parameterization models of the stoichiometric process.^16^ Some studies have reported that high atomic number material, such as barium (Z = 56), is not appropriate for stoichiometric CT number calibration.^16^, ^17^ A conventional stoichiometric CT number calibration is performed using nominal CT numbers for air (−1000 HU) and water (0 HU). However, there are CT scanners in which the CT number for air is not −1000 HU,^18^, ^19^ so, as fitting parameters in a conventional stoichiometric CT number calibration are forcibly determined, using the CT number −1000 HU for air, a CT number calibration error may be introduced — particularly for low density tissues such as lung. In a separate issue, a required minimum number of materials for stoichiometric CT number calibration has not been established — and this number needs to be known for downsizing the CT number calibration audit phantom for the postal audit.

The purpose of this study was to establish a CT number calibration audit, for photon radiation therapy, to validate patient imaging and calibration processes applied via third parties. As described in the first part of this article (Sections 2.A and 2.B), we attempted to develop a new stoichiometric CT number calibration, determine a suitable set of materials, and identify the minimal number of materials appropriate for stoichiometric CT number calibration — and then to verify our methods by comparing measured and theoretical CT numbers. As described in the second part of this article (Sections 2.C and 2.D), we designed a CT number calibration audit phantom, and validated actual CT number calibration tables registered in RTPSs with the audit phantoms, for multiple institutions.

## 2 MATERIAL AND METHODS

### 2.1 Development of a new stoichiometric CT number calibration

Scheneider et al.^8^ established a stoichiometric CT number calibration to convert CT numbers directly to MDs and elemental weights for Monte Carlo dose calculation. Their method applied three steps:

1. CT scans of a set of materials with known MDs and elemental weights were conducted.
2. A multiparameter fit between the MDs, elemental weights, and the measured CT numbers was established.
3. Theoretical CT numbers with obtained parameters, known MDs, and elemental weights were calculated.

The CT numbers were defined as shown in Eq. (1):

(1) $$H=1000\left(\frac{\mu }{\mu_{H_{2}O}}-1\right),$$

where *H* denotes the CT number, and $\mu /\mu_{H_{2}O}$ is the ratio of the linear attenuation coefficient relative to water. Fraction $\mu /\mu_{H_{2}O}$ is defined with two free parameters, and known MD and elemental weights, as shown in Eq. (2):

(2) $$\frac{\mu }{\mu_{H_{2}O}}\left(k_{1},k_{2}\right)=\frac{\rho }{\rho_{H_{2}O}}\frac{\sum_{i}\left(w_{i}/A_{i}\right)\left(Z_{i}+Z_{i}^{2.86}k_{1}+Z_{i}^{4.62}k_{2}\right)}{\left(w_{H}/A_{H}\right)\left(1+k_{1}+k_{2}\right)+\left(w_{O}/A_{O}\right)\left(8+8^{2.86}k_{1}+8^{4.62}k_{2}\right)},$$

where $\rho /\rho_{H_{2}O}$ is the ratio of the MD relative to water; *i* is the element index; *w_i_*, *A_i_*, and *Z_i_* are the elemental weight, atomic mass, and atomic number of index *i*, respectively. Symbols *w*_H_ and *A*_H_ represent the elemental weight and atomic mass of hydrogen, respectively; *w*_O_ and *A*_O_ are the elemental weight and atomic mass of oxygen, respectively; *k*_1_ and *k*_2_ represent the free parameters experimentally determined by performing a least square fit with the measured CT numbers. The least square fit derived from Eqs. (1) and (2) is as shown in (3), where *n* is the material index:

(3) $$\sum_{n}{\left[{\left(\frac{\mu }{\mu_{H_{2}O}}\left(k_{1},k_{2}\right)\right)}_{n}-{\left(\frac{H}{1000}+1\right)}_{n}\right]}^{2}.$$

This least square fit is a two‐parameter fit model. The initial parameters of *k*_1_ and *k*_2_ were given as 1.24 × 10^−3^ and 3.06 × 10^−5^, respectively.^8^ The theoretical CT numbers were then calculated, by substituting the resultant values for *k*_1_ and *k*_2_ into Eq (4):

(4) $$H_{t}=1000\left(\frac{\rho }{\rho_{H_{2}O}}\frac{\sum_{i}\left(w_{i}/A_{i}\right)\left(Z_{i}+Z_{i}^{2.86}k_{1}+Z_{i}^{4.62}k_{2}\right)}{\left(w_{H}/A_{H}\right)\left(1+k_{1}+k_{2}\right)+\left(w_{O}/A_{O}\right)\left(8+8^{2.86}k_{1}+8^{4.62}k_{2}\right)}-1\right),$$

where *H*_t_ is the theoretical CT number. Air and water CT numbers, as calculated using Eq. (4), are always equal to −1000 HU and 0 HU, respectively. However, the CT number for air is not always equal to −1000 HU,^18^, ^19^ and varies depending on the scan parameter.^19^ To harmonize the theoretical CT number with the measured CT number for air, we established a new stoichiometric CT number calibration, with an empirical three‐parameter fit, by adding a parameter, *α*, to Eq. (1), as follows:

(5) $$H=1000\alpha \left(\frac{\mu }{\mu_{H_{2}O}}-1\right),$$

where *α* is a free parameter, with the nominal value of one. Values for *k*_1_, *k*_2_, and *α* were determined by performing a least square fit with the measured CT numbers. The least square fit applying Eqs (2) and (5) is as follows:

(6) $$\sum_{n}{\left[{\left(\frac{\mu }{\mu_{H_{2}O}}\left(k_{1},k_{2}\right)\right)}_{n}-{\left(\frac{H}{1000\alpha }+1\right)}_{n}\right]}^{2}.$$

The initial values for *k*_1_, *k*_2_, and *α* were 1.24 × 10^−3^, 3.06 × 10^−5^, and 1.0, respectively.^8^ This least square fit is a three‐parameter fit model, and the theoretical CT numbers were calculated by substituting the resultant values for *k*_1_, *k*_2_, and *α* into Eq. (7):

(7) $$H_{t}=1000\alpha \left(\frac{\rho }{\rho_{H_{2}O}}\frac{\sum_{i}\left(w_{i}/A_{i}\right)\left(Z_{i}+Z_{i}^{2.86}k_{1}+Z_{i}^{4.62}k_{2}\right)}{\left(w_{H}/A_{H}\right)\left(1+k_{1}+k_{2}\right)+\left(w_{O}/A_{O}\right)\left(8+8^{2.86}k_{1}+8^{4.62}k_{2}\right)}-1\right).$$

The parameter fit was automatically performed with the general programming language Python, and the open‐source, SciPy (http://www.scipy.org) Python package was used to minimize Eqs. (3) and (6).

**Figure 1:**
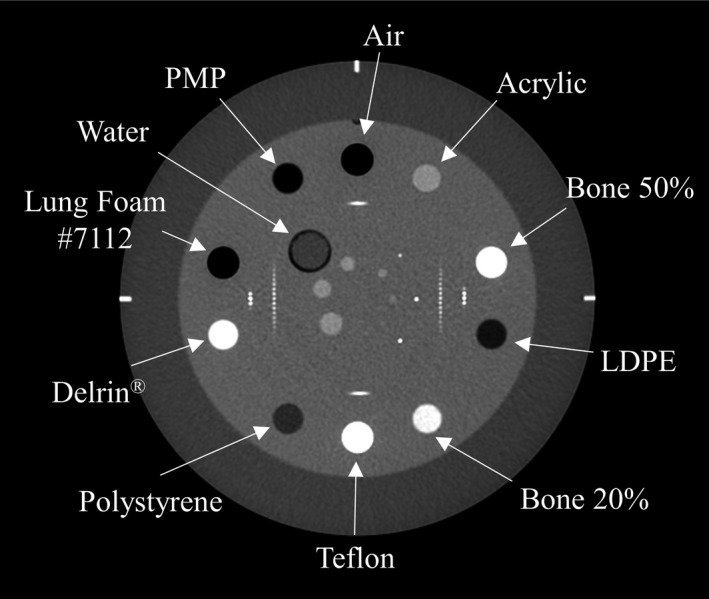
CT image of the Catphan 700 phantom (CTP682), into which 11 sensitometric materials had been inserted.

To compare the three‐parameter fit model with the conventional two‐parameter fit model, a new stoichiometric CT number calibration benchmark was performed, using Catphan 700 (CTP682) (The Phantom Laboratory, Salem, NY, USA) for multiple CT images. Catphan 700 (CTP682) was scanned by several CT scanners, under various scan conditions and at multiple institutions (Fig. 1). The phantom diameter was 200 mm, and included 11 sensitometric materials, whose MDs and material weights were provided by the manufacturer.

We experimented with performing a least square fit with a selection of the 11 sensitometric materials, to determine the fewest number of materials that could be used to capture adequate elemental information. The selection of materials has been summarized in Table 1, and the “All materials and elements” group is all the materials and elements used for a least square fit. The characteristics of the “a number of materials” group were that the number of materials used was large, while the number of elements contained in them was small. The characteristics of the “a number of elements” group were that two materials were used, while the number of elements contained in them was larger than was contained overall in the “a number of materials” group.

**Table 1:** MDs and elements of materials inserted into the Catphan 700 (CTP682) phantom. Three material groups were used for a least square fit in the stoichiometric CT number calibration.

Material	ρ [g/cm^3^]	Element	All materials and elements group	A number of materials group	A number of elements group
Air	1.33 × 10^−3^	N, O, Ar	✓	✓	
Lung #7112	1.76 × 10^−1^	H, C, N, O	✓	✓	✓
PMP	8.30 × 10^−1^	H, C	✓	✓	
LDPE	9.20 × 10^−1^	H, C	✓	✓	
Water	1.00	H, O	✓	✓	
Polystyrene	1.03	H, C	✓	✓	
Bone 20%	1.14	H, C, N, O, P, Ca	✓		
Acrylic	1.18	H, C, O	✓	✓	
Bone 50%	1.40	H, C, N, O, P, Ca	✓		✓
Delrin^®^	1.42	H, C, O	✓	✓	
Teflon	2.16	C, F	✓		

MD, mass density.

The fitting parameters obtained from these three material groups were used to calculate theoretical CT numbers for 11 sensitometric materials. The theoretical CT numbers were then compared to the measured CT numbers, using 14 CT images obtained from six CT scanners located at five radiotherapy institutions. The characteristics of the 14 CT images have been summarized in Table 2.

**Table 2:** Summary of scan conditions for 14 CT images obtained from six CT scanners in at five radiotherapy institutions.

Location	CT scanner	Tube voltage (kV)	Tube current (mA)	Slice thickness (mm)	Acquisition field of view (mm)	Reconstruction field of view (mm)	Reconstruction filter
A	GE LightSpeed RT 16	120	200	2.5	250	250	STANDARD
		120	200	2.5	500	500	STANDARD
B	Toshiba Asteion TSX‐021A	120	200	2.0	320	320	FC10
		120	200	2.0	500	480	FC10
C	Toshiba Aquilion LB	120	350	2.0	240	240	FC21
		120	350	2.0	320	320	FC21
		120	126	2.0	400	400	FC03
D	GE Optima CT 580 W	80	300	2.5	500	500	STANDARD
		100	300	2.5	500	500	STANDARD
		120	300	2.5	500	500	STANDARD
		140	300	2.5	500	500	STANDARD
E	GE HiSpeed NXI	120	66	5.0	500	500	STD+
	GE Optima CT 580 W	120	330	2.5	500	300	STANDARD
		120	53	2.5	500	500	STANDARD

### 2.2 Materials selection for the new stoichiometric CT number calibration

In this study, lung equivalent material (tough lung) and bone equivalent material (tough bone) (Kyoto Kagaku, Kyoto, Japan) were used for the new stoichiometric CT number calibration. The insert size was 2 cm in diameter and 4 cm in length, and the MDs and elemental weights have been listed in Table 3. The elemental weights of H, C, and N were analyzed by performing a quantitative analysis using a CHNS‐O 2400II PARKIN‐ELMER elemental analyzer (PerkinElmer Inc., Waltham, MA, USA). Other elemental weights, except for H, C, N, and O were analyzed by performing a semi‐quantitative analysis, using a RIX 2000 X‐ray fluorescence analyzer (Rigaku, Akishima, Japan).

**Table 3:** MDs and elemental weights for the Kyoto Kagaku phantom: MDs and elemental weights for tough water, tough lung, and tough bone were obtained from the manufacturer, as indicated. The additional MDs and elemental weights for tough lung and tough bone were measured using two analyzers, for stoichiometric CT number calibration.

	Tough water (Manufacturer)	Tough lung (Manufacturer)	Tough bone (Manufacturer)	Tough lung (Measurement)	Tough bone (Measurement)
ρ [g/cm^3^]	1.018	0.370	1.500	0.360	1.495
*w*_H_ [%]	8.63	7.00	5.11	6.01	5.29
*w*_C_ [%]	68.89	50.20	42.45	63.41	42.73
*w*_N_ [%]	2.18	–	1.73	0.37	1.96
*w*_O_ [%]	17.88	35.10	28.13	29.16	29.42
*w*_Na_ [%]	–	–	–	0.10	0.56
*w*_Mg_ [%]	–	–	–	–	0.08
*w*_Al_ [%]	–	1.50	7.00	0.18	–
*w*_Si_ [%]	–	5.00	–	0.67	0.03
*w*_P_ [%]	–	0.10	–	0.01	6.01
*w*_S_ [%]	–	–	–	0.01	0.02
*w*_Cl_ [%]	0.15	1.00	0.09	0.03	0.29
*w*_K_ [%]	–	–	–	0.03	0.05
*w*_Ca_ [%]	2.27	–	15.49	0.01	13.54
*w*_Fe_ [%]	–	–	–	0.01	–
*w*_Sr_ [%]	–	–	–	–	0.01
*w*_unknown_ [%]	–	–	–	–	0.01

MD, mass density.

When the total weight was defined as 100%, the elemental weight of O was calculated as a residual weight, because it was not analyzed using both of the analyzers. The MDs were obtained by dividing the weight by the volume, according to ISO‐845.^20^

**Figure 2:**
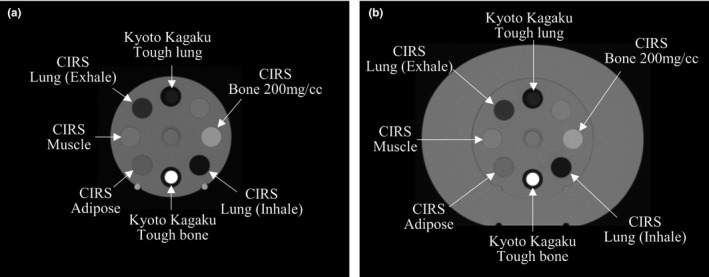
Transverse slices through the CIRS 062M phantom: (a) head phantom; (b) body phantom. The seven materials (five from the CIRS 062M, and tough lung and bone from Kyoto Kagaku) were scanned simultaneously, using the same scanner.

To validate the new stoichiometric CT number calibration with tough lung and tough bone, the theoretical CT numbers from the CIRS 062M (CIRS Model 062M electron density phantom, CIRS, Inc., Norfolk, VA, USA) were compared with the measured CT numbers. Five tissue‐equivalent materials in the CIRS 062M — lung (inhale), lung (exhale), adipose, muscle, and bone 200 mg/cc — were scanned using a GE Optima CT 580 W (GE Medical Systems, Milwaukee, WI, USA), and the CT numbers were measured. At the same time, tough lung and tough bone from Kyoto Kagaku were scanned (Fig. 2), and a least square fit was performed, using the formula for a three‐parameter fit minimizing model Eq. (6), with the MDs and elemental weights, and the measured CT numbers from the Kyoto Kagaku phantom.

The theoretical CT numbers from the CIRS 062M were calculated with the obtained three parameters, MDs, and elemental weights provided by the manufacturer.^16^ Air and bone 800 mg/cc from the CIRS 062M were also scanned with a CT scanner, to create the CT‐RED table for air and bone 800 mg/cc. The theoretical CT number for bone 800 mg/cc was not calculated in this study because bone 800 mg/cc contains high atomic number materials, such as barium (Z = 56).^16^

The theoretical and measured CT numbers from the CIRS 062M were compared for head and body phantoms, as CT numbers vary depending on phantom size. The CT numbers were obtained by averaging the CT numbers in the region of interest (ROI) from one CT image, and the diameter of the ROI circle was smaller than that of the insert circle.

### 2.3 Design of the CT number calibration audit phantom

**Figure 3:**
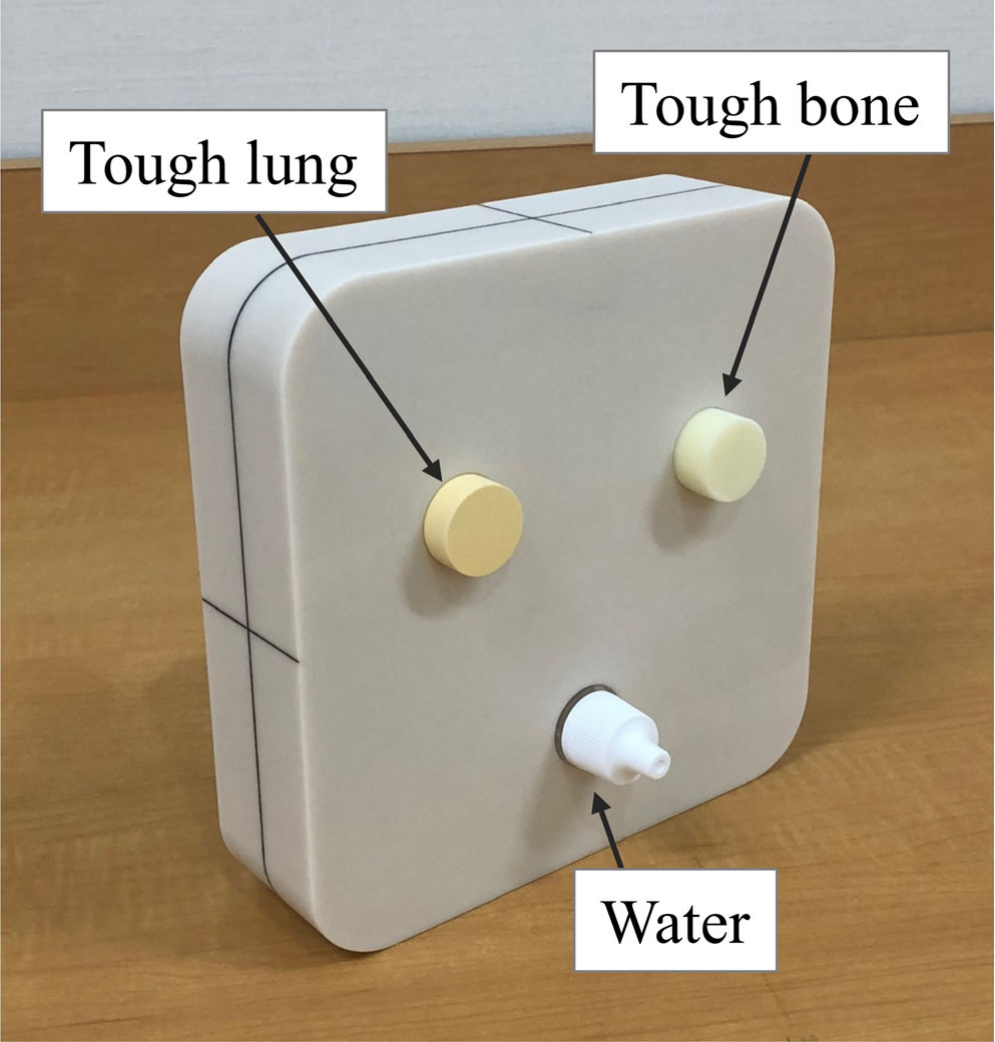
CT number calibration audit phantom. [Color figure can be viewed at http://wileyonlinelibrary.com]

We then designed a CT number calibration audit phantom (Fig. 3). A postal audit phantom should be small and hard to break; therefore, in this case, the dimensions of the phantom were 150 mm × 150 mm × 40 mm, and its body consisted of a water‐equivalent material (tough water) (Kyoto Kagaku, Kyoto, Japan). The tough lung, tough bone, and tough water were inserted in a circle, with the center of the circle coinciding with the center of the phantom. The tough lung, bone, and water were then scanned with a CT scanner.

The measured CT numbers for tough lung and tough bone were used to perform a three‐parameter fit, using the measured CT numbers, MDs, and elemental weights minimizing formula, Eq. (6) — and the MDs and elemental weights have been listed in Table 3. The CT number for water was not used to perform the three‐parameter fit, as $\mu /\mu_{H_{2}O}$ is always one, for Eq. (2) — and therefore, the CT number for water always equals zero in Eq. (7), for new stoichiometric CT number calibration. In order to verify the water CT number through measurement, a water target was inserted in the CT number calibration audit phantom.

CT‐MD and CT‐RED calibration tables were created using 11 representative tissues, in this study. The 11 representative tissues were defined by Kanematsu et al.,^21^ and classified using MDs from adult reference computational phantom data, from ICRP 110.^12^ As the human body consists of the six major elements, M = {H, C, N, O, P, Ca}, the residual weight, and the mean residual atomic numbers of the other minor elements (Na, Mg, S, Cl, K, Fe, and I) were calculated as shown in Eq. (8):

(8) $$w_{\text{res}}=\sum_{r\notin M}w_{r},\;and\;{\bar{Z}}_{\text{res}}=\frac{\sum_{r\notin M}Z_{r}w_{r}}{w_{\text{res}}},$$

where *w*_res_ is the weight of the residual element; *w_r_* and Z*_r_* are the weight and atomic number of residual element *r*, respectively; and ${\bar{Z}}_{\text{res}}$ is the mean atomic number of the residual elements. The atomic mass of residual element A_res_ is approximately 2.1×${\bar{Z}}_{\text{res}}$, in the human body.

The MDs, REDs, major elements, residual element weights, and mean residual atomic numbers for the 11 representative tissues have been summarized in Table 4. The theoretical CT numbers for the 11 representative tissues were calculated by applying Eq. (7), using the three obtained parameters, *k*_1_, *k*_2,_ and *α*, and the tissue data, and were compared to the measured CT numbers obtained using the CIRS 062M and Gammex 467 (Gammex 467 Tissue Characterization Phantom, Gammex Inc., Middleton, WI, USA) phantoms. In this study, CIRS 062M values were scanned using a CT scanner with a body‐sized phantom.

**Table 4:** MDs, REDs, major elements, residual element weights, and mean residual atomic numbers for 11 representative tissues^21^.

Name	*ρ *[g/ cm^3^]	*ρ*_e_ */ (ρ*_e_ *)*_w_	*w*_H_ [%]	*w*_C_ [%]	*w*_N_ [%]	*w*_O_ [%]	*w*_P_ [%]	*w*_Ca_ [%]	*w*_res_ [%]	${\bar{Z}}_{\text{res}}$
Air	0.001	0.001	0.00	0.01	75.52	23.17	0.00	0.00	1.30	18.0
Lung	0.384	0.381	10.3	10.7	3.2	74.6	0.2	0.0	1.0	15.9
Extra Lung	0.80	0.79	10.3	10.7	3.2	74.6	0.2	0.0	1.0	15.9
Fat	0.90	0.91	11.96	76.87	0.00	11.17	0.00	0.00	0.00	–
Adipose/ Marrow	0.950	0.951	11.40	58.92	0.74	28.64	0.00	0.00	0.30	14.7
Muscle/ General	1.049	1.040	10.25	14.58	3.20	70.87	0.21	0.02	0.87	16.8
Miscellaneous	1.090	1.077	9.94	20.90	3.84	63.73	0.45	0.27	0.87	15.5
Heavy Spongiosa	1.136	1.115	9.30	39.15	2.22	41.71	2.36	4.60	0.66	14.9
Mineral Bone	1.92	1.784	3.6	15.9	4.2	44.8	9.4	21.3	0.8	13.1
Tooth	2.75	2.518	2.2	9.5	2.9	42.1	13.7	28.9	0.7	12.0
Hydroxyapatite	3.156	2.830	0.20	0.00	0.00	41.14	18.50	39.89	0.00	–

MD, mass density; RED, relative electron density to water.

### 2.4 CT number calibration audit for multiple institutions

The CT number calibration audit phantom was scanned using six CT scanners located at five radiotherapy institutions. The five radiotherapy institutions, six CT scanners, own tissue‐equivalent phantoms, and CT number calibration table types (CT‐RED or CT‐MD) have been summarized in Table 5. The scan conditions, including tube voltage, reconstruction filter, acquisition field of view, slice thickness, and tube current, were the same as those that applied to the treatment planning CT scan.

**Table 5:** Summary of five radiotherapy institutions, six CT scanners, this study’s tissue‐equivalent phantom, and CT number calibration types registered in the RTPSs.

Location	CT scanner	Tissue equivalent material	CT number calibration type
A	GE LightSpeed RT 16	Gammex 467	CT‐MD and CT‐RED calibration
B	Toshiba Asteion TSX‐021A	Gammex 467	CT‐MD calibration
C	Toshiba Aquilion LB	Gammex 467	CT‐RED calibration
D	GE Optima CT 580 W	CIRS 062M	CT‐MD and CT‐RED calibration
E	GE Optima CT 580 W GE HiSpeed NXI	CIRS 062M	CT‐RED calibration

CT numbers for water, tough lung, and tough bone were measured, and the measured CT number for water was compared to its theoretical counterpart, namely zero, while the measured CT numbers for tough lung and tough bone were used for the new stoichiometric CT number calibration. To evaluate differences between our stoichiometric CT number calibration table and the tables registered in the RTPSs, MD, and RED differences were calculated, by subtracting the numbers in the CT number calibration table registered in the RTPS from those from our table. The results were then compared to the tolerance levels for each tissue type,^22^, ^23^ and the CT number calibration table was then classified by the MD range, according to the tolerance level definition. The MD ranges for lung, adipose/ muscle, and cartilage/ spongy‐bone were 0.2–0.8 g/cm^3^, 0.9–1.07 g/cm^3^, and 1.07–1.25 g/cm^3^, respectively.

## 3 RESULTS

### 3.1 Development of a new stoichiometric CT number calibration

**Figure 4:**
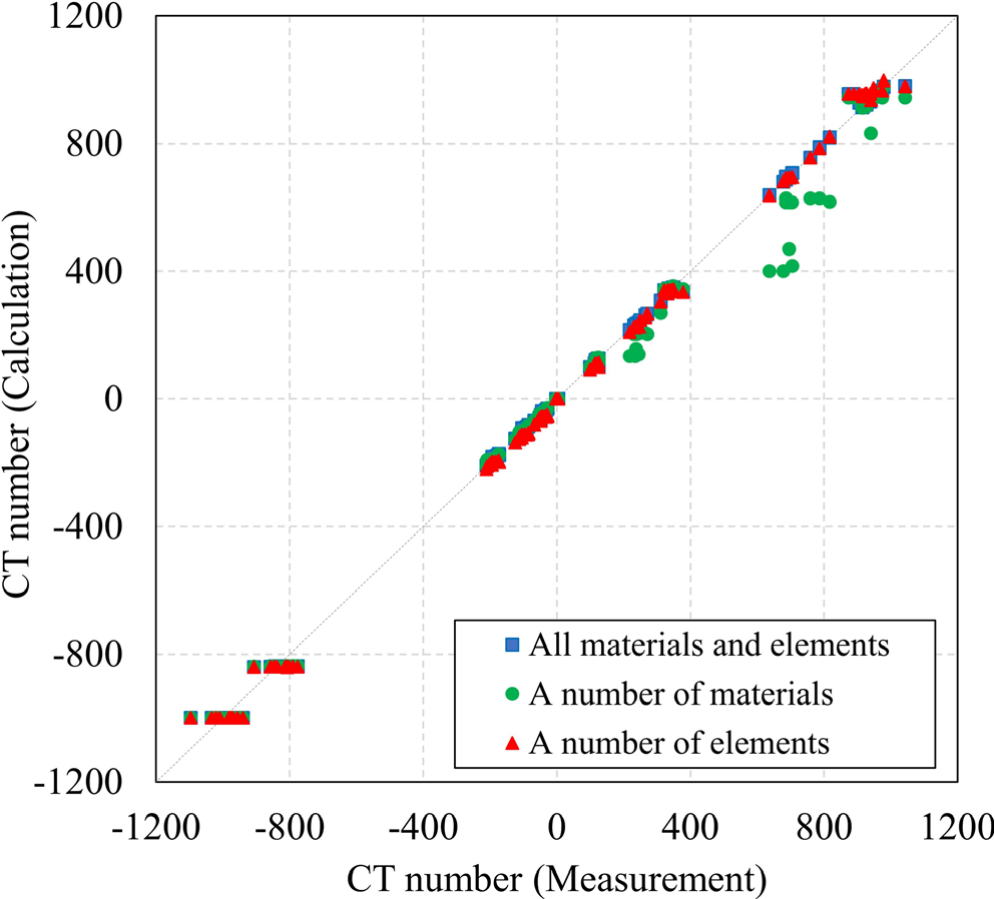
Comparison between measured and theoretical CT numbers, calculated using Eq. (4), with a two‐parameter fit model, for the Catphan 700 (CTP682) phantom. The dashed line represents the ideal case, where calculated values equal measurements. [Color figure can be viewed at http://wileyonlinelibrary.com]

A comparison between the measured and theoretical CT numbers has been prepared as Figure 4, calculated using Eq. (4), with a two‐parameter fit model for the Catphan 700 (CTP682) phantom. The theoretical CT numbers were compared to the measured CT numbers for each of the 14 CT images from the six CT scanners. Assuming that the theoretical CT numbers from the “all materials and elements” group mostly corresponded with the measured CT numbers, the theoretical CT numbers of the “a number of elements” group corresponded with the measured CT numbers better than those from the “a number of materials” group. The theoretical CT numbers for air and lung #7112 did not correspond with the measured CT numbers for any material groups, since the theoretical CT number for air was fixed as −1000 HU.

**Figure 5:**
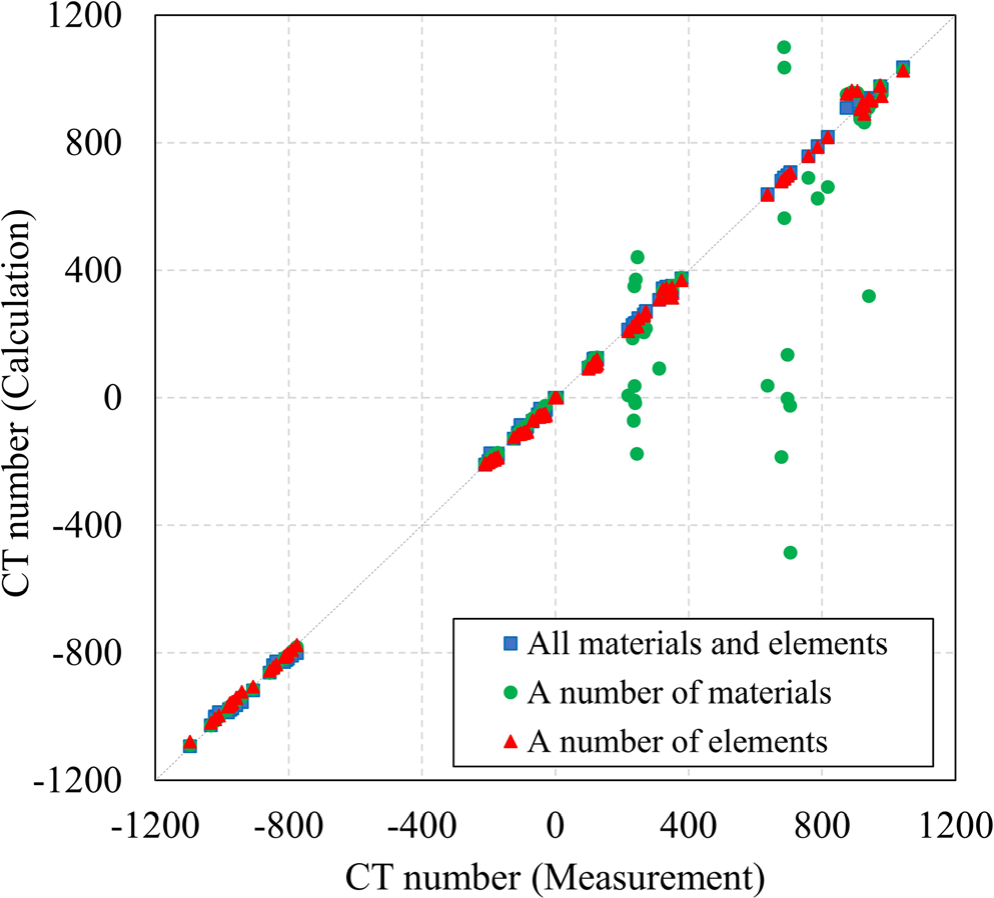
Comparison between measured and theoretical CT numbers, calculated using Eq. (7), with the three‐parameter fit model, for the Catphan 700 (CTP682) phantom. The dashed line represents the ideal case, where calculated values equal measurements. [Color figure can be viewed at http://wileyonlinelibrary.com]

Figure 5 shows the comparison between the measured and theoretical CT numbers, calculated with Eq. (7), and using the three‐parameter fit model. The theoretical CT numbers for the three‐parameter fit model corresponded with the measured CT numbers better than those of the two‐parameter fit model, for air and lung #7112, because the theoretical CT numbers for air are described as −1000*α* HU, according to Eq. (5). The difference between the theoretical and measured CT numbers of the “a number of materials” group was increased by adding the free parameter, *α*, while the difference between the theoretical and measured CT numbers for the “a number of elements” group was similar to that for the “all materials and elements” group.

### 3.2 Materials selection for the new stoichiometric CT number calibration

**Figure 6:**
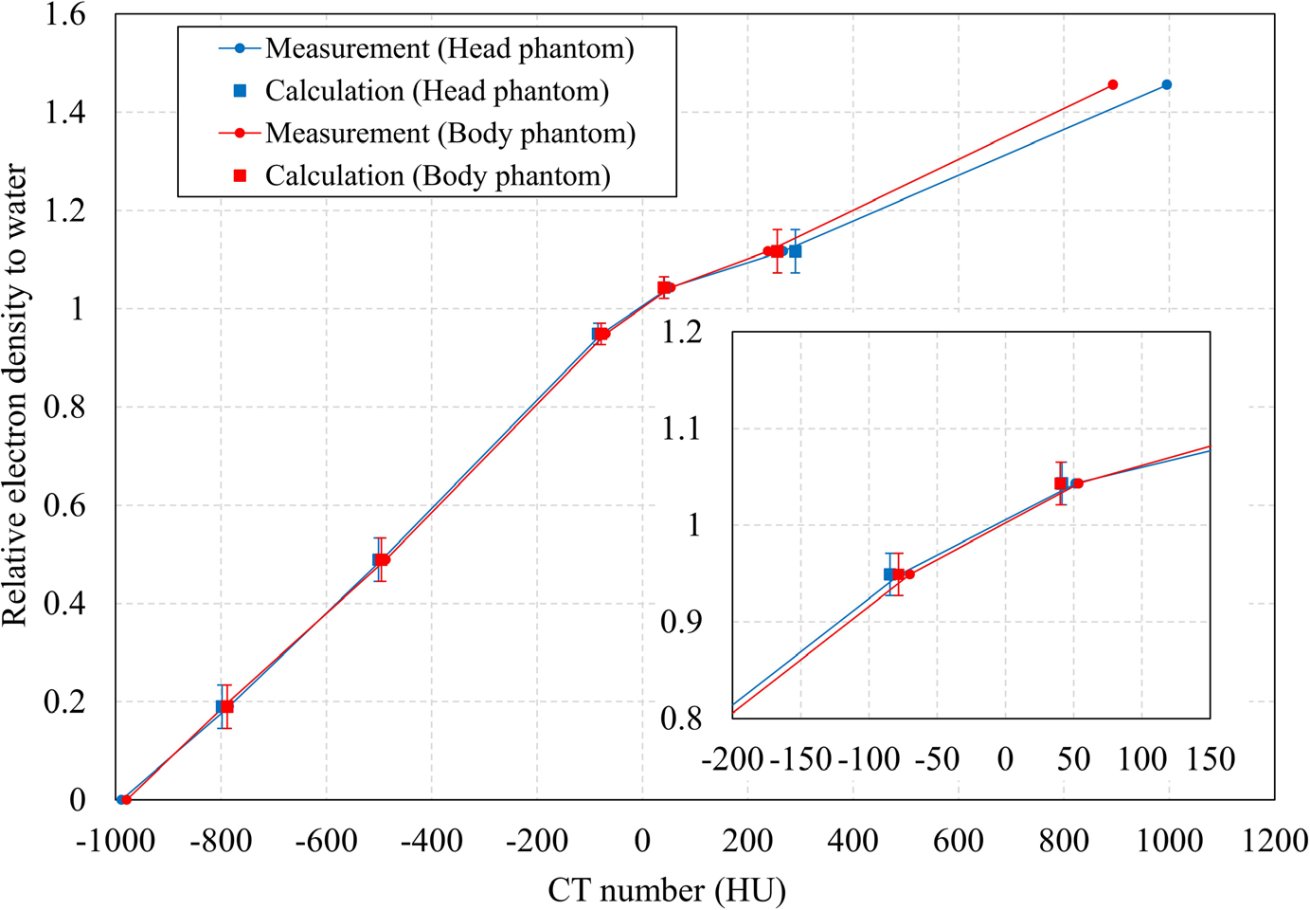
Comparison between theoretical and measured CT numbers for the CIRS 062M phantom. The bars are the tolerance levels for each tissue type,^22^ and correspond to a 2% dose difference. [Color figure can be viewed at http://wileyonlinelibrary.com]

The new stoichiometric CT number calibrations for tough lung and tough bone were compared with the tissue‐substitute calibration of CIRS 062M for the head and body phantoms (Fig. 6). The bars are the tolerance levels for each tissue type, which correspond to a 2% dose difference.^22^ The tolerance levels were ±0.044, ±0.022, and ±0.044, for lung, adipose/ muscle, and cartilage/spongy‐bone, respectively. The differences between the theoretical and measured CT numbers were <1% of the dose difference (one‐half of the tolerance levels^22^) for the same phantom size. The theoretical CT numbers for bone 800 mg/ cc from the CIRS 062M phantom were excluded, since bone 800 mg/cc contains barium, and there was a large difference between the calculation and the measurement.^16^ The RED difference for phantom size increased between −200 HU and −50 HU, while above 200 HU, however, this difference was less than the tolerance levels for adipose/ muscle and cartilage/ spongy‐bone.^22^

### 3.3 Design of the CT number calibration audit phantom

**Figure 7:**
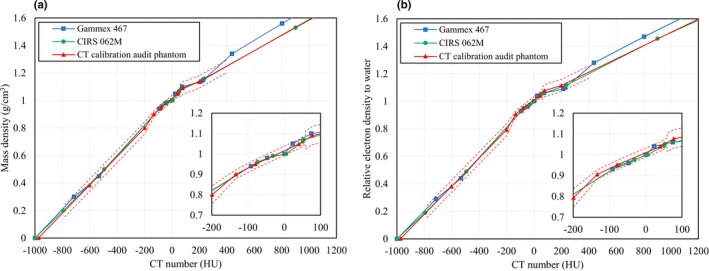
Comparisons between tissue‐substitute calibrations using a commercially available tissue‐equivalent phantom and the stoichiometric CT number calibration established using the audit phantom: (a) CT‐MD calibration; (b) CT‐RED calibration. The dashed lines represent the theoretical CT number calibration table ± tolerance level^22^, ^23^. [Color figure can be viewed at http://wileyonlinelibrary.com]

Theoretical CT numbers for 11 representative tissues were calculated with a three‐parameter fit model, using Eq. (7), and the MDs and REDs for these tissues were used to create CT‐MD and CT‐RED calibration tables. The theoretical CT number calibration tables created with the audit phantom were compared to the measured CT number calibration tables obtained using commercially available tissue‐equivalent phantoms — CIRS 062M and Gammex 467 (Fig. 7). The measured CT number calibration tables were obtained using a body‐sized phantom.

The CIRS 062M phantom was 330 mm wide and 270 mm high, the Gammex 467 was 330 mm in diameter, while the CT number calibration audit phantom was 150 × 150 mm^2^. When the same scan conditions were used, the differences between the CT number calibration audit phantom and CIRS 062M were less than the tolerance levels,^22^, ^23^ for both the CT‐MD and CT‐RED calibrations. The difference between the CT number calibration audit phantom and the Gammex 467 slightly exceeded the MD tolerance level, for cartilage/ spongy‐bone.

### 3.4 CT number calibration audit for multiple institutions

**Figure 8:**
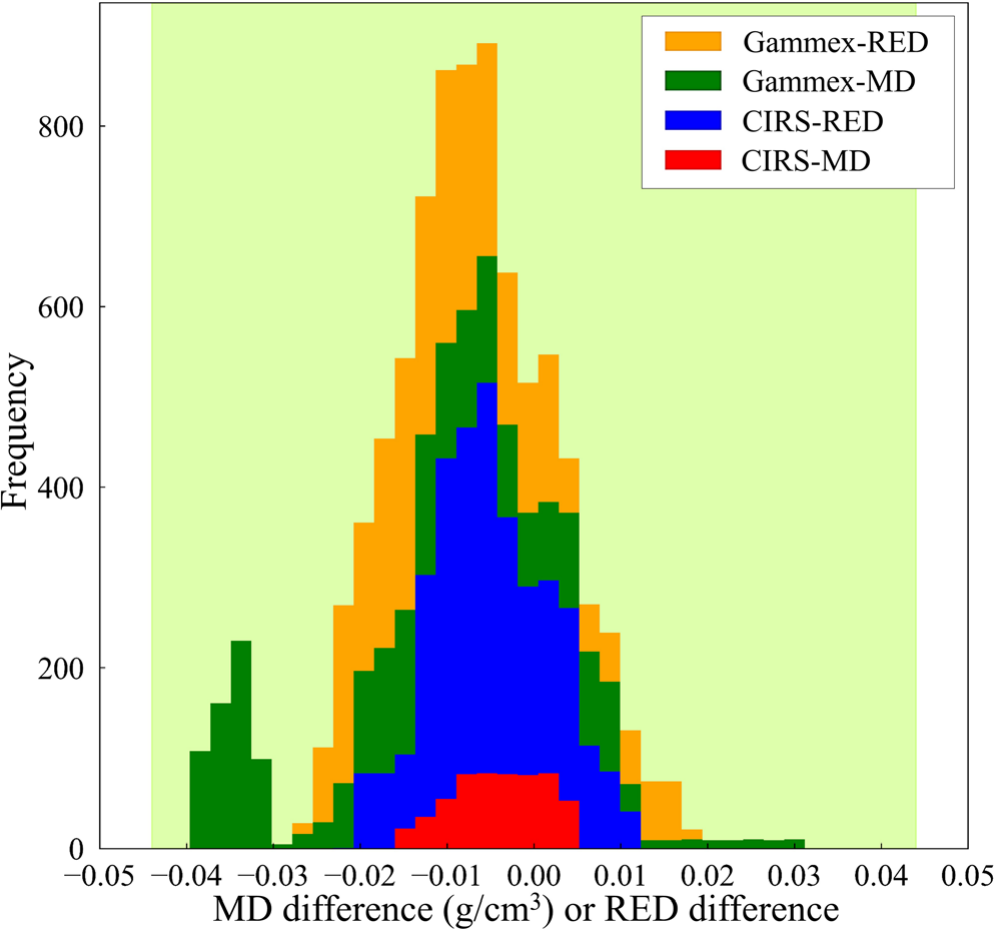
A stacked histogram for each CT number calibration type, for lung. The tolerance level range is 0 ± 0.044. [Color figure can be viewed at http://wileyonlinelibrary.com]

**Figure 9:**
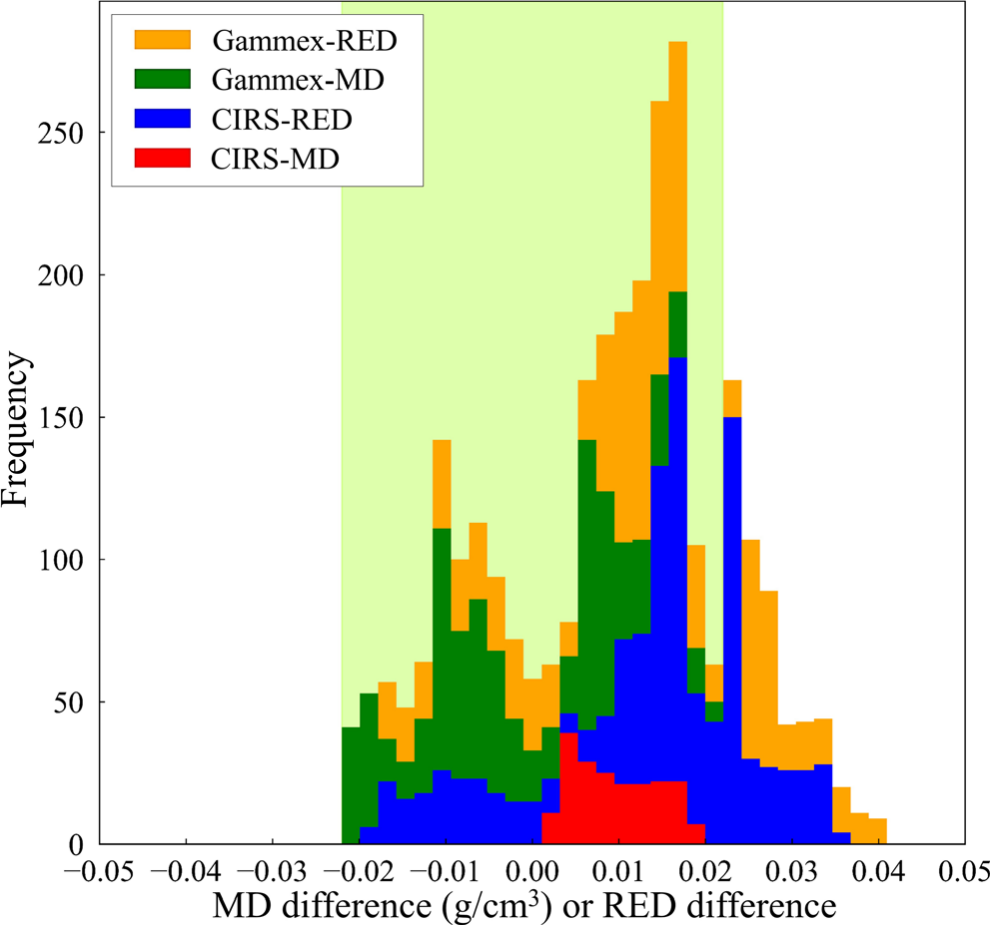
A stacked histogram for each CT number calibration type, for adipose/ muscle. The MD range is 0.9–1.07 g/cm^3^. The tolerance level range is 0 ± 0.022. [Color figure can be viewed at http://wileyonlinelibrary.com]

**Figure 10:**
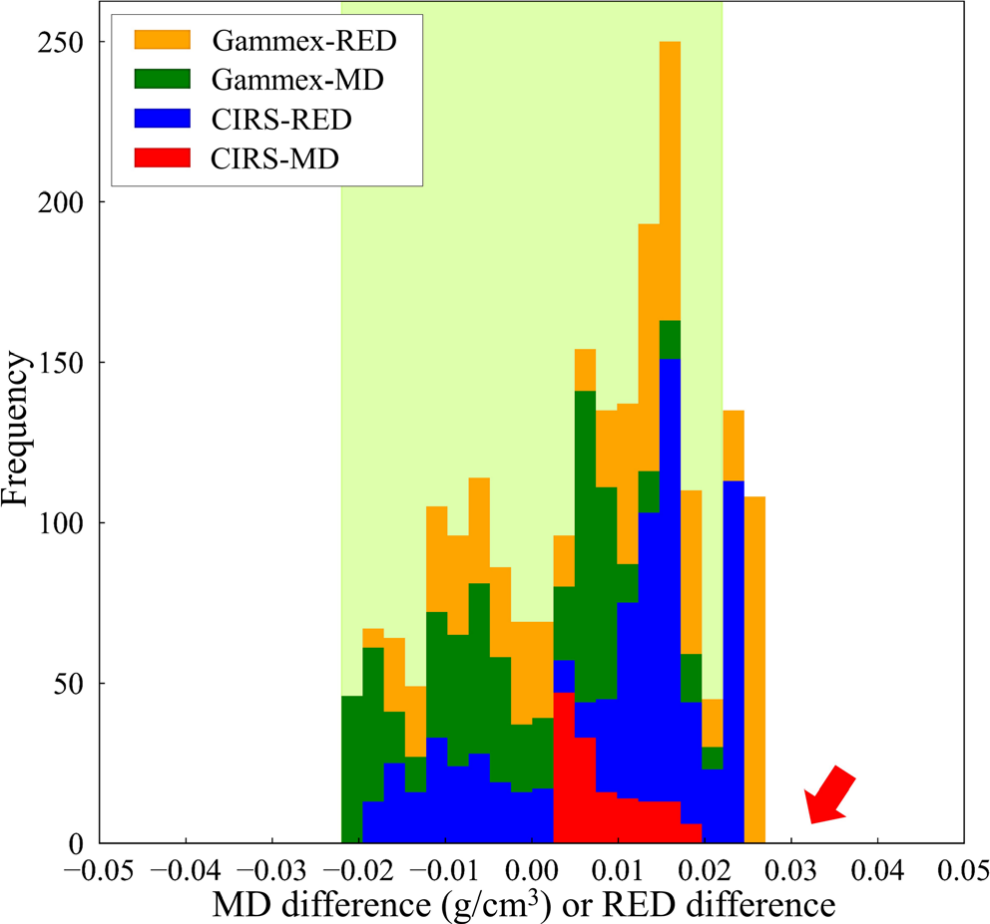
A stacked histogram for each CT number calibration type, for adipose/ muscle. The MD range is 0.95–1.07 g/cm^3^. The tolerance level range is 0 ± 0.022. The arrow shows the decreasing change, from 0.90–1.07 g/cm^3^ to 0.95–1.07 g/cm^3^. [Color figure can be viewed at http://wileyonlinelibrary.com]

**Figure 11:**
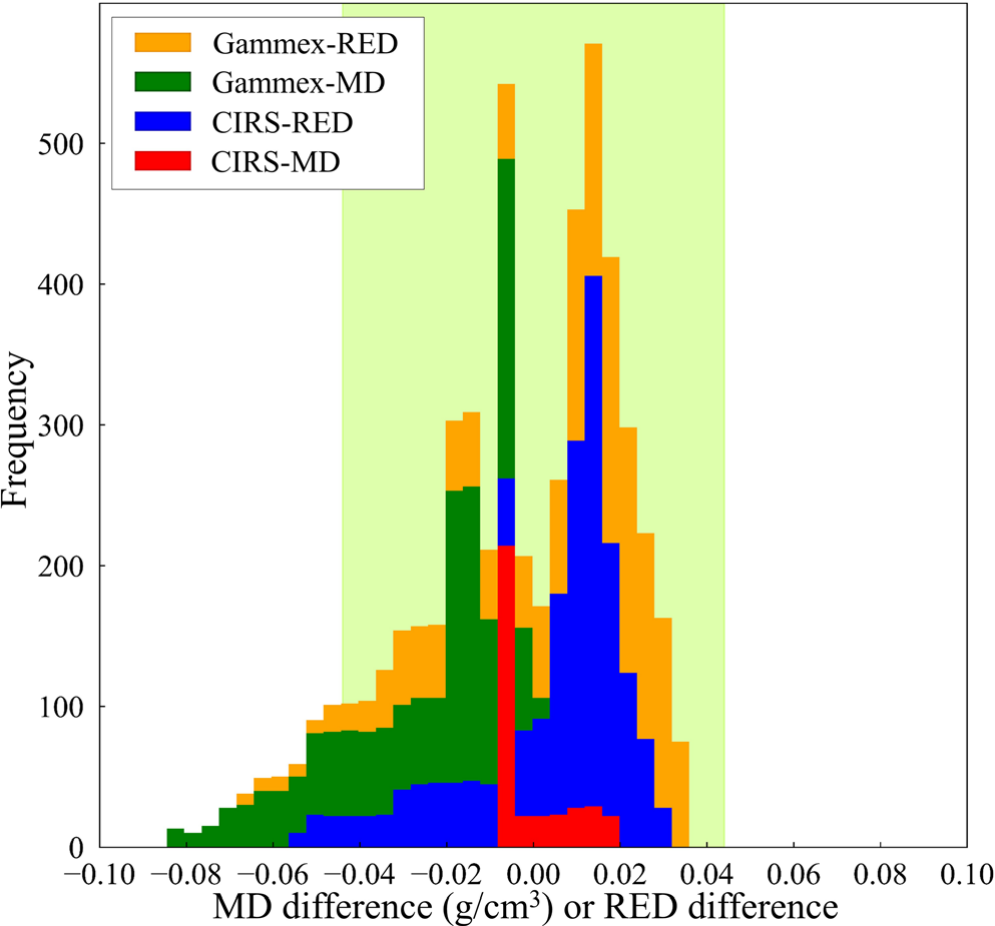
A stacked histogram for each CT number calibration type, for cartilage/ spongy‐bone. The tolerance level range is 0 ± 0.044. [Color figure can be viewed at http://wileyonlinelibrary.com]

Table 6 shows the scan conditions and CT numbers for water, for all CT scanners, at multiple institutions, and it can be seen that the measured CT numbers for water were all within ±5 HU. The theoretical CT number calibration tables with the audit phantom were compared to the CT number calibration tables registered in the RTPSs, and the differences for each CT number calibration type are shown in Figs. 8, 9, 10, 11, as stacked histograms. The CT number calibration type was determined using the RTPS and the dose calculation algorithm. The stacked histograms were categorized as Gammex‐RED, Gammex‐MD, CIRS‐RED, and CIRS‐MD, using the CT number calibration type and own tissue‐equivalent phantom. The MD and RED differences were stacked in the same histogram, as the difference between the MD and RED tolerance levels was <0.001.^23^ Table 7 shows the MD and RED differences for each tissue type, each tolerance level, CT number calibration type, and own tissue‐equivalent phantom.

**Table 6:** Summary of CT scanner type, scan conditions, and CT numbers for water, for multiple institutions

Location	CT scanner	Tube voltage (kV)	Tube current (mA)	Slice thickness (mm)	Acquisition field of view (mm)	Reconstruction field of view (mm)	Reconstruction filter	CT number of water Mean ± SD (HU)
A	GE LightSpeed RT 16	120	250	2.5	250	250	STANDARD	2.8 ± 4.0
		120	300	2.5	500	500	STANDARD	2.6 ± 6.9
B	Toshiba Asteion TSX‐021A	120	200	2.0	320	320	FC10	1.3 ± 5.1
		120	200	2.0	500	480	FC10	3.3 ± 6.4
C	Toshiba Aquilion LB	120	350	2.0	240	240	FC21	−1.1 ± 4.4
		120	250	2.0	320	320	FC21	−2.3 ± 5.8
		120	250	2.0	400	400	FC03	−0.9 ± 5.6
D	GE Optima CT 580 W	120	400	1.25	500	500	STANDARD	0.0 ± 3.2
E	GE HiSpeed NXI	120	247	3.0	500	300	STD+	1.0 ± 2.8
		120	247	3.0	500	500	STD+	0.7 ± 3.2
	GE Optima CT 580 W	120	321	1.25	500	300	STANDARD	1.3 ± 4.8
		120	321	1.25	500	500	STANDARD	0.6 ± 3.6

**Table 7:** MD and RED differences for each tissue type, each tolerance level, own tissue‐equivalent phantom, and each CT number calibration type.

Tissue type	Mass density range	Tolerance level	Tissue equivalent phantom ‐ CT number calibration type	MD and RED differences Mean ± SD
Lung	0.2–0.8 g/cm^3^	±0.044	Gammex‐RED	−0.008 ± 0.010
			Gammex‐MD	−0.013 ± 0.016 g/cm^3^
			CIRS‐RED	−0.005 ± 0.007
			CIRS‐MD	−0.004 ± 0.005 g/cm^3^
Adipose/muscle	0.9–1.07 g/cm^3^	±0.022	Gammex‐RED	0.012 ± 0.013
			Gammex‐MD	−0.001 ± 0.011 g/cm^3^
			CIRS‐RED	0.014 ± 0.013
			CIRS‐MD	0.010 ± 0.005 g/cm^3^
Cartilage/spongy‐bone	1.07–1.25 g/cm^3^	±0.044	Gammex‐RED	0.005 ± 0.023
			Gammex‐MD	−0.026 ± 0.020 g/cm^3^
			CIRS‐RED	0.004 ± 0.019
			CIRS‐MD	0.000 ± 0.008 g/cm^3^

MD, mass density; RED, relative electron density to water.

The MD and RED differences for lung are shown in Fig. 8, and the MD or RED differences were less than the tolerance level (±0.044) for lung.

The MD and RED differences for adipose/muscle are shown in Fig. 9. Some RED differences exceeded the tolerance level (±0.022) for adipose/ muscle, at the rate of 27.1% of the total RED difference. The MD range for adipose/ muscle was 0.9–1.07 g/cm^3^, with the lower border identified as 0.9 g/cm^3^ fat (Table 4). Although the RED of fat is 0.91, the figures for the CIRS 062M and Gammex 467 phantoms were 0.949 and 0.930, respectively.

Figure 10 shows the MD and RED differences for adipose/muscle when the MD range was 0.95–1.07 g/cm^3^. The rate, which exceeded the tolerance level, decreased from 27.1% to 17.3% of the total RED difference when the MD range was changed from 0.9–1.07 g/cm^3^ to 0.95–1.07 g/cm^3^.

The MD and RED differences for cartilage/ spongy‐bone are shown in Fig. 11. A part of the MD or RED differences exceeded the relevant tolerance level (±0.044), and the rates were 3.7%, 21.9%, and 3.2%, for the Gammex‐RED, Gammex‐MD, and CIRS‐RED phantoms, respectively.

## 4 DISCUSSION

Conventional stoichiometric CT number calibration uses the two‐parameter fit model of the relationship between the MD, material weight, and measured CT number. We have established a new stoichiometric CT number calibration scheme, using an empirical, three‐parameter fit model. The new scheme was benchmarked using a Catphan 700 (CTP682) phantom, which gave us adequate elemental information for benchmark testing, as the inserted materials contained 2–6 elements. If materials contain >six elements, the least number of materials required could not be determined using this benchmark. Assuming that the result of a least square fit using all materials was highly accurate, we investigated how many materials needed to be used, and the number of elements that needed to be contained in them, for an acceptable stoichiometric CT number calibration.

The stoichiometric CT number calibration was established based on the cross‐sectional parameterization given by Rutherford et al.^24^ Cross‐sectional parameterization is described with an atomic number and three coefficients — for photoelectric effect, coherent scattering, and incoherent scattering. Two free parameters, namely *k*_1_ and *k*_2_, are related to the coefficients in the cross‐sectional parameterization.

The experimental work reported in Sections 2.A and 3.A made it clear that the atomic number is critical for automatic parameter fit in a least square fit process. Although the “a number of materials” group contained eight materials, between them they consisted of just five elements — H, C, N, O, and Ar. This indicated that *k*_1_ and *k*_2_ were not determined with five elements alone. By then comparing the “a number of elements” group with the “a number of materials” group, we were able to show that the result of a least square fit was determined not by the number of materials used, but by the number of elements that they contained (Figs. 4 and 5).

Stoichiometric CT number calibration was therefore performed using only two materials, with these containing six elements. In addition, when least square fitting was performed for all materials using multiple CT scanners, it was shown that for several of the scanners, the measured and theoretical CT numbers were inconsistent for air and lung tissue, in the conventional stoichiometric CT number calibration.

The difference between the measured and theoretical CT numbers was caused by the theoretical CT number for air being fixed as −1000 HU in the conventional stoichiometric CT number calibration. The measured CT number for air was different for each CT scanner manufacturer and scan parameters^18^, ^19^— and the measured air CT number range was −1100 to −920 HU. Although the CT scanners used in this study were calibrated daily, in free air (air calibration), the CT number for air inside the phantom differed from the nominal value of −1000 HU because the substantial phantom was not used when air calibration was performed. In particular, the measured CT number for air was different inside and outside the phantom, with the number measured outside being closer to the nominal −1000 HU value.^18^

The CT numbers measured inside the phantom are more relevant for dose calculation; therefore, in this study, a new parameterization model was established by adding one empirical parameter, *α*, in which the theoretical CT number of air has been described as −1000*α* HU, to the conventional stoichiometric CT number calibration. Once the three‐parameter fit model had been established in Section 2.A and 3.A, this was the fit model used in Sections 2.2, 2.4, and 3.2, 3.4.

Tough lung and tough bone were selected for the CT number calibration audit phantom, based on the benchmarking referred to above. These phantoms were suitable, as they had more than six elements, and did not contain high atomic number materials such as barium (Z = 56). Although the MDs and elemental weights for tough lung and tough bone were provided by the manufacturer, we confirmed these measurements using an electronic balance and two elemental analyzers, as MD and elemental weight accuracy are critical for stoichiometric CT number calibration (Table 3).

It can be seen in Table 3 that there were several differences between manufacturer specifications and our experimental measurements; for example, tough lung MD showed a difference of 2.7%. In addition, trace element information was not provided by the manufacturer, and Na, Mg, S, K, and Sr, were detected in our experimental measurements through X‐ray fluorescence. All elemental weights, including those of the trace elements, were used for stoichiometric CT number calibration, while the MD and elemental weight for tough water were not measured, as they were not used for stoichiometric CT number calibration.

To verify the stoichiometric CT number calibration performance for tough lung and tough bone, theoretical CT numbers for tissue‐equivalent materials in the CIRS 062M phantom were calculated, based on the measured CT numbers for tough lung and tough bone. The theoretical and measured CT numbers for the tissue‐equivalent materials from the CIRS 062M phantom were compared, because the tissue‐equivalent materials of CIRS 062M had known MDs and elemental weights.^16^ This meant that, as the difference between the theoretical and measured CT numbers was <1% dose difference for the same phantom size, the selection of phantoms, measured MDs, and analyzed elemental weights could be deemed suitable for performing stoichiometric CT number calibration.

Although the measured CT number difference greater than 200 HU increased when using the different‐sized phantom, the REDs difference was less than the tolerance levels (which corresponded to a 2% dose difference). Therefore, it was considered that a small‐sized phantom (150 × 150 × 40 mm^3^) was appropriate for use in validating the CT number calibration table obtained using the body size phantom.

A prototype CT number calibration audit phantom was designed, and the CT number calibration table was compared with that of commercially available tissue‐equivalent phantoms, for multiple institutions. The theoretical CT number calibration tables were calculated with obtained parameters and standard tissue data, based on ICRP 110,^12^ meaning that the theoretical CT number table was comparable to that for standard tissue.

The CT number differences from different‐sized phantoms have been shown in Fig. 6. Here it could be seen that the measured CT numbers >200 HU increased when the small‐sized phantom was used.

The curves in Fig. 7 showed CT number differences by phantom size, for the higher CT number range. The results showed that although the CIRS 062M phantom was much larger than the CT number calibration audit phantom (330 mm × 270 mm × 50 mm) vs (150 mm × 150 mm × 40 mm), the MD and RED differences were both <the tolerance levels for each tissue type. Also, the CIRS 062M curves matched those of the CT calibration audit phantom, for the higher CT number range.

Bone 800 mg/cc in the CIRS 062M phantom contained high atomic number material, such as barium (Z = 56), which has an elemental weight of 0.28. Except for barium, bone 800 mg/cc incorporates natural human tissue components, including H, C, N, O, P, S, Cl, and Ca. Therefore, for both the MD and RED of bone 800 mg/cc, it could be assumed that the measured CT number agreed with the theoretical CT number for standard tissue.

Part of the MD difference between the CT number calibration audit phantom and the Gammex 467 (330 mm diameter × 50 mm) phantom exceeded the cartilage/ spongy‐bone tolerance level. This difference showed the same trend as that exhibited in the differences between the different‐sized phantoms in Fig. 6. Another contributing factor was the difference between the Gammex 467 tissue‐equivalent material and standard tissue, because 30% CaCO_3_ (ρ = 1.34 g/cm^3^) and 50% CaCO_3_ (ρ = 1.56 g/cm^3^) were used to create the CT‐MD and CT‐RED calibration tables, respectively. A previous study^13^ reported that REDs for bone substitute phantoms greater than 200 HU were higher than those of real tissues examined under ICRU 44^10^ and ICRU 46^11^— and our work gave the same outcome.

Finally, the stoichiometric CT number calibration tables with the audit phantom were compared to the CT number calibration tables registered in the RTPSs of multiple institutions. When CT number calibration audits are performed with tissue‐equivalent materials, it is difficult to select suitable options. Data for many candidates were available from ICRP 23,^9^ ICRU 44,^10^ ICRU 46,^11^ and ICRP 110,^12^ and all must be scanned and compared with CT calibration tables. On the other hand, our stoichiometric method is useful for the CT number calibration audit activity, as the CT numbers for all standard tissues can be calculated by scanning just two materials.

The real water value was inserted into the CT number calibration audit phantom, to verify the CT number accuracy for water. For all CT scanners in Table 6, numbers for water were within 0 ± 5 HU.^25^

The MD and RED differences for lung were less than the tolerance level (±0.044) for all CT number calibration types.

The RED difference in the adipose/ muscle sample partially exceeded the tolerance level, and it was assumed that this difference (from 0.90–0.95 g/cm^3^) exceeded the tolerance level because the rate decreased from 27.1% to 17.3% of the total RED difference when the MD range was changed from 0.9–1.07 g/cm^3^ to 0.95–1.07 g/cm^3^. MDs for subcutaneous and internal fat, at 37℃, were 900.0 ± 1.03 kg/m^3^, and 900.0 ± 5.1 kg/m^3^, respectively.^26^ The MDs for commercially available adipose equivalent materials are between 0.94 and 0.96 g/cm^3^; however, the difference between the tissue‐substitute calibration and the stoichiometric CT number calibration increased, from between 0.90 and 0.95 g/cm^3^, because 0.9 g/cm^3^ was selected as the lower border for adipose/ muscle in this study.

Considering the results for cartilage/ spongy‐bone (Fig. 11), the difference between Gammex‐MD and standard tissue analyses was the greatest of the four factors (Table 7). The MD difference was compared to the tolerance level, exhibiting a 2% dose difference; however, the dose difference occurred at the 10‐cm thickness point, in a 10 cm × 10 cm field^22^. This suggests that, even if part of the histogram indicated tolerance level exceedance, the 2% dose difference was not caused by the CT number calibration error. On the other hand, if the mean MD or RED difference value exceeded the tolerance level, it could be assumed that a CT number calibration error had occurred.

The American Association of Physicists in Medicine Task Group 85 (AAPM TG‐85)^27^ recommended 2% accuracy in tissue inhomogeneity correction, by accepting that the overall accuracy should be 5% for dose delivery. Relative dose calculation requires two steps: (a) establishing a calculation in a homogeneous medium, and (b) applying a tissue inhomogeneity correction. The proposed audit in this study validated the CT number calibration table — applying the MD and RED tolerance levels that caused a 2% dose error — using the effective depth inhomogeneity correction algorithm.^22^, ^23^, ^28^ If an uncertainty of 2% in tissue inhomogeneity correction is divided evenly, each of these independent components would have to show an uncertainty of <1.4%. The modified tolerance levels causing 1.4% dose errors were calculated to be 0.031, 0.015, and 0.031, for lung, adipose/ muscle, and cartilage/ spongy‐bone, respectively.

In this study, actual CT number calibration tables registered in the RTPSs were validated for multiple institutions using the audit phantom, with the results summarized in Table 7. The MD and RED difference means, for all tissue‐equivalent phantom CT number calibration types, were less than the modified tolerance levels that caused 1.4% dose errors. This indicated that the modified tolerance levels noted above could be considered as suitable tolerance levels for the CT number calibration audit.

Limitations to this work include the issue that a small audit phantom (150 × 150 × 40  mm^3^) was used for the CT number calibration audit. A small phantom is suitable for the postal audit; however, CT numbers vary with phantom size, indicating that phantom size should be carefully determined, by reviewing the required tolerance levels.^22^, ^23^

In a second issue, the tissue‐substitute CT number calibration has the problem of forcibly using tissue‐equivalent material supplied by the phantom manufacturers. This meant that the CT number calibration error increased when the difference between the tissue‐equivalent material and standard tissue increased. On the other hand, conventional stoichiometric CT number calibration has the problem of an increasing CT number calibration error that occurs when the difference between the measured and nominal CT numbers for air increases. The new stoichiometric CT number calibration using the three‐parameter fit model should be able to resolve both of these problems.

## 5 CONCLUSIONS

We established a new stoichiometric CT number calibration method, using a three‐parameter fit model, and developed a CT number calibration audit phantom — and verified their performance using CT scanners located in several institutions. Our stoichiometric CT number calibration system has the advantages of needing to use only two materials, and decreasing the differences between theoretical and measured CT numbers for air and lung tissues. To validate the patient imaging and calibration processes of third parties, a postal CT number calibration audit should be achievable in the future, using a small‐sized phantom.

## CONFLICT OF INTEREST

The authors have no conflicts of interest.
